# Acupotomy for third lumbar vertebrae transverse process syndrome: A protocol for systematic review

**DOI:** 10.1097/MD.0000000000021072

**Published:** 2020-07-17

**Authors:** Lin Jiao, Yuanyi Xiao, Zhenhai Chi, Daocheng Zhu, Xilin Ouyang, Wei Xu, Jianyu You, Zhaona Luo, Fuqiang Yuan

**Affiliations:** a Jiangxi University of Traditional Chinese Medicine; b Affiliated Hospital of Jiangxi University of Traditional Chinese Medicine, Nanchang, China.

**Keywords:** acupotomy, protocol, systematic review, third lumbar vertebrae transverse process syndrome

## Abstract

**Background:**

Acupotomy has been widely used clinically to relieve low back pain. However, the efficacy of acupotomy for the third lumbar vertebrae transverse process syndrome is still uncertain. The aim of this study is to determine the effectiveness and safety of acupotomy therapy for the third lumbar vertebrae transverse process syndrome.

**Methods:**

Relevant randomized controlled trials will be searched from the databases of PubMed, the Cochrane Library, Embase, the China National Knowledge Infrastructure, Wanfang Database, Chinese Science and Technology Periodical Database, and Chinese Biomedical Literature Database from their inception to May 2020. Two reviewers will independently select studies, collect data, and assess the methodology quality by the Cochrane risk of bias tool. The RevMan V.5.3 will be used for meta-analysis.

**Results:**

This study will provide an assessment of the current state of acupotomy for the third lumbar vertebrae transverse process syndrome, aiming to show the efficacy and safety of acupotomy treatment.

**Conclusion:**

This study will provide evidence to judge whether acupotomy is an effective intervention for the third lumbar vertebrae transverse process syndrome.

**PROSPERO registration number:**

CRD42019134945.

## 1 Introduction

Low back pain (LBP) is a widespread public health problem. It is the leading cause of activity limitation and absenteeism from work,^[1,2]^ and results in a huge economic cost and medical burden.^[3,4]^ According to the Global Burden of Disease Study, back pain is one of the leading causes of disability in the world.^[5]^ According to statistics, the global prevalence of LBP among adults is about 12%, and the lifetime prevalence is about 40%.^[6]^ In addition, as people's lifestyles change and the population ages, recent reports indicate that this prevalence may increase.^[6,7]^ Among the causes of LBP, the third lumbar vertebrae transverse process syndrome is one of the common causes. The third lumbar vertebra is the center of vertebral rotation and forward bending, and has multiple muscle attachments, so the contraction of the lumbar spine and abdomen may cause mechanical stress at the apex of the transverse process of the third lumbar vertebra, resulting in acute injury and chronic strain. Long-term mechanical stress stimulation eventually leads to the formation of the third lumbar vertebrae transverse process syndrome.^[8]^

Regarding the treatment management of the third lumbar vertebrae transverse process syndrome, the current treatment methods mainly involve pharmacological and nonpharmacological measures. First-line drug therapy usually includes acetaminophen or NSAID. Although most patients can improve symptoms through drug therapy, the use of drug therapy is limited by adverse reactions.^[7,9]^ In addition, it is reported that nonpharmacological methods such as psychotherapy, osteopathy, multidisciplinary rehabilitation, physical exercise, and massage also have certain effects, but due to the small number of people used, the level of evidence is not high, and the clinical application still exists dispute.^[7,10]^ Therefore, there is an urgent need to find a safer and more effective alternative therapy.

Acupotomy is a new minimally invasive treatment method that combines scalpel and acupuncture. It uses acupuncture theory as the guiding ideology, and absorbs modern pathology and anatomy theory as well as anesthesia and aseptic technique,^[11]^ it can not only achieve the stimulation effect of acupuncture, but also play the role of cutting and peeling of scalpel, which can effectively eliminate adhesion, reduce the tension of soft tissue, and restore the normal function of the tissue,^[12]^ and has the characteristics of small wound, high safety, and high treatment efficiency.^[13]^ Therefore, it is widely used clinically in musculoskeletal diseases including the third lumbar vertebrae transverse process syndrome.^[14,15]^

Although the benefit of acupotomy has been widely proven, the effectiveness of acupotomy treatment of the third lumbar vertebrae transverse process syndrome is still controversial. Therefore, this study uses the method of evidence-based medicine to analyze and evaluate the randomized controlled trials (RCTs) of patients with third lumbar transverse process syndrome treated by acupotomy, and provides a basis for further improving the clinical efficacy.

## 2 Methods

### 2.1 Inclusion criteria for study selection

#### 2.1.1 Types of studies

All RCTs of acupotomy for the third lumbar vertebrae transverse process syndrome will be included without language restriction. Non-RCTs, observational studies, cross-over studies, uncontrolled trials, animal trials, and reviews will be excluded.

#### 2.1.2 Types of participants

Inclusion criteria for study populations will be all patients with third lumbar vertebrae transverse process syndrome. No restrictions will be applied in terms of gender, age, race, condition duration, or intensity.

#### 2.1.3 Types of interventions

##### 2.1.3.1 Experimental interventions

The treatment group will only receive acupotomy therapy alone, without any restrictions on needle material, shape, or treatment process.

##### 2.1.3.2 Control interventions

The control group will receive an internationally recognized therapy such as pharmacological therapies. Placebo, no treatment, and acupuncture will also be included. Studies that compare the effect of different types of acupotomy will be excluded.

#### 2.1.4 Types of outcome measures

##### 2.1.4.1 Primary outcomes

Visual analogue scale (VAS) and Percentage of Clinical Effectiveness will be accepted as the primary outcomes.

##### 2.1.4.2 Additional outcomes

The safety assessment will be considered a secondary outcome.

### 2.2 Search methods for the identification of studies

#### 2.2.1 Electronics searches

The following electronic databases will be searched: PubMed, Embase, the Cochrane Library, the China National Knowledge Infrastructure, Chinese Science and Technology Periodical Database, Wanfang Database, and Chinese Biomedical Literature Database. We will search the databases from the beginning to May 2020. Search terms consist of disease (third lumbar vertebrae transverse process syndrome, third lumbar transverse process syndrome, third lumbar, low back pain) and intervention (acupotomy, needle knife, acupotomology, needle scalpel) and research types (randomized controlled trial, controlled clinical trial, random trials). The PubMed search strategy is shown in Table 1.

**Table 1:**
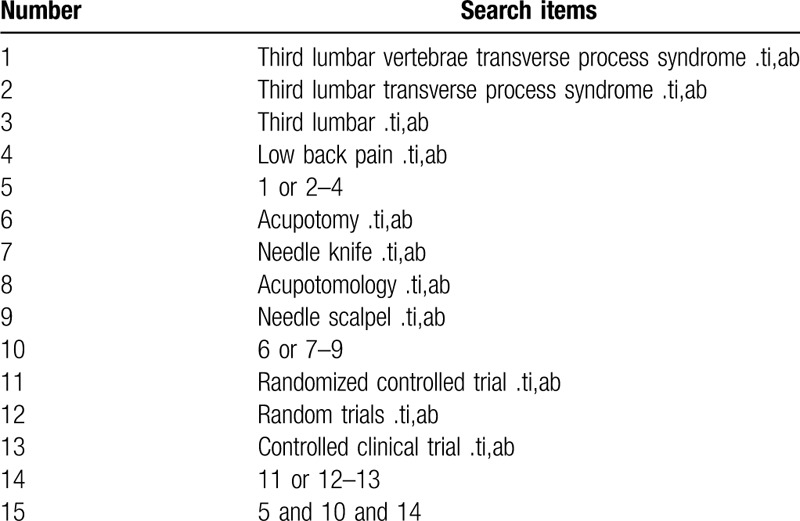
Search strategy used in PubMed database.

#### 2.2.2 Search for other resources

We will also retrieve the relevant conference papers, and search for new trials related to acupotomy treatment of the third lumbar vertebrae transverse process syndrome on the WHO International Clinical Trials Registration Platform (ICTRP) and the Clinical Trials.gov.

### 2.3 Data collection and analysis

#### 2.3.1 Selection of studies

**Figure 1:**
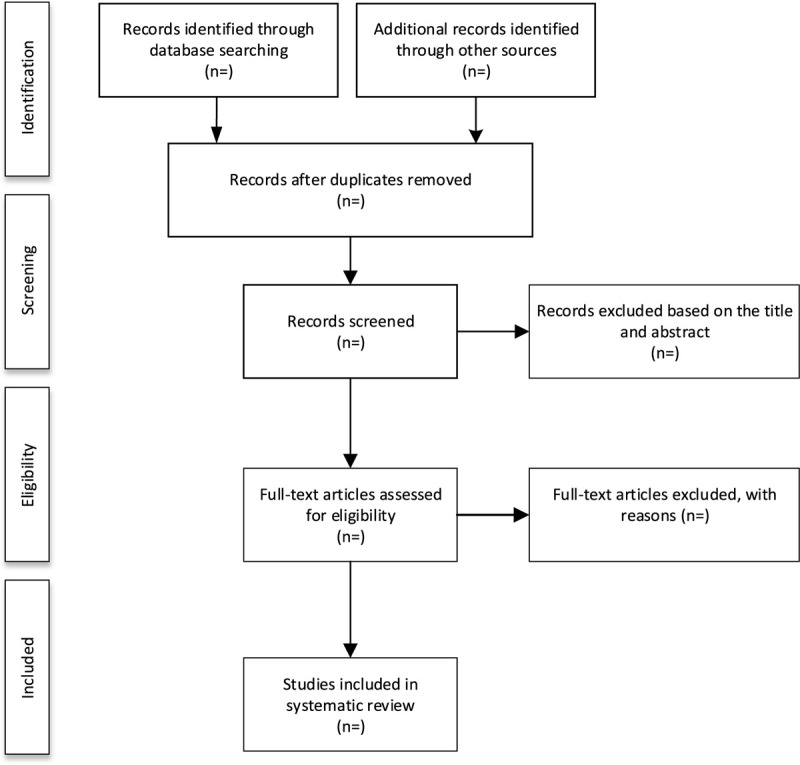
Flow diagram of study selection process.

We will import the retrieved literature into EndNote X7 software and delete the duplicate data. After that, 2 reviewers will independently scan the titles and abstracts. Unrelated literature will be deleted. If they cannot determine whether to include the study, they will obtain the full text of the article for judgment. Two reviewers will independently evaluate the eligibility of these articles based on inclusion and exclusion criteria. Any disagreements will be resolved through group discussions. The study selection procedure is shown in Figure 1.

#### 2.3.2 Data extraction and management

The data extraction for eligible studies will be completed independently by 2 authors, and any disagreement will be resolved through discussion with the third author. The extracted data will mainly include the first author, time of publication, patient characteristics, sample size, interventions, follow-up period, outcome measures, and adverse events. If necessary, we will try to contact the author for the details by email.

### 2.4 Risk of bias assessment

Two independent authors will evaluate the risk of bias among the final included studies using the risk of bias assessment tool by the Cochrane Collaboration.^[16]^ The contents will include: random sequence generation; allocation concealment; blinding of participants and personnel; blinding of outcome assessment; incomplete outcome data; selective reporting; and other sources of bias. Each study will be evaluated as High, Low, or Unclear risk of bias for each item. Discrepancies will be resolved through further discussion with the third author.

### 2.5 Quantitative data synthesis and statistical methods

#### 2.5.1 Quantitative data synthesis

We will conduct statistical analysis through RevMan 5.3 software. For categorical data, we will calculate with the risk ratio (RR) and 95% confidence intervals (CIs). For continuous variables, mean difference (MD) will be included in the meta-analysis. If outcome variables are measured on different scales, results will be reported as standardized mean differences (SMDs) with 95% CI.

#### 2.5.2 Assessment of heterogeneity

We will use *χ*^2^ test and *I*^*2*^ test to evaluate the statistical heterogeneity. When *P* > .10 and *I*^*2*^ ≤ 50%, the research results will not be considered heterogeneous; otherwise, it will be considered as heterogeneous.

#### 2.5.3 Assessment of reporting biases

When more than 10 studies are included, funnel plot will be generated to detect the reporting bias. In addition, we will use the Egger test to check the asymmetry of funnel plot.

#### 2.5.4 Subgroup analysis

If the included studies have significant heterogeneity, we will perform subgroup analysis based on different control groups.

#### 2.5.5 Sensitivity analysis

When sufficient studies are available, sensitivity analysis will be used to assess the robustness of the meta-analysis based on methodological quality, sample size, and missing data.

#### 2.5.6 Grading the quality of evidence

We will assess the quality of evidence by the Grading of Recommendations Assessment, Development and Evaluation and rate it into high, moderate, low or very low 4 levels.^[17,18]^

## 3 Discussion

Acupotomy has been widely used in the treatment of the third lumbar vertebra transverse process syndrome. Related clinical studies have shown that acupotomy can effectively relieve the symptoms of the third lumbar vertebra transverse process syndrome.^[14,15]^ In addition, related basic experimental studies have shown that acupotomy can reduce pain by regulating inflammation, and can promote the recovery of soft tissue function,^[19,20]^ but the clinical efficacy of acupotomy has not been scientifically and systematically evaluated. The aim of this study is to evaluate the clinical efficacy and safety of acupotomy treatment for the third lumbar vertebrae transverse process syndrome. The conclusions drawn by this study may provide evidence-based medical advice for the treatment of the third lumbar vertebrae transverse process syndrome with acupotomy. However, this study may also have some potential limitations. First, during acupotomy treatment, the choice of treatment site, the time and frequency of operation may be heterogeneous. Second, the reliability of the systematic review largely depends on the comprehensiveness and methodological quality of the studies included in this review.

## Author contributions

**Data curation:** Lin Jiao, Yuanyi Xiao, Zhenhai Chi.

**Formal analysis:** Yuanyi Xiao, Fuqiang Yuan.

**Investigation:** Yuanyi Xiao, Zhenhai Chi.

**Methodology:** Yuanyi Xiao, Fuqiang Yuan.

**Project administration:** Lin Jiao.

**Software:** Yuanyi Xiao, Zhenhai Chi.

**Supervision:** Lin Jiao.

**Validation:** Lin Jiao.

**Visualization:** Yuanyi Xiao.

**Writing – original draft:** Yuanyi Xiao, Zhenhai Chi.

**Writing – review & editing:** Lin Jiao, Fuqiang Yuan.
